# Automatic breast lesion segmentation in phase preserved DCE-MRIs

**DOI:** 10.1007/s13755-022-00176-w

**Published:** 2022-05-20

**Authors:** Dinesh Pandey, Hua Wang, Xiaoxia Yin, Kate Wang, Yanchun Zhang, Jing Shen

**Affiliations:** 1 Victoria University, Melbourne, Australia; 2 Guangzhou University, Guangzhou, China; 3 RMIT University, Melbourne, Australia; 4 Radiology Department, Affiliated Zhongshan Hospital of Dalian University, Dalian, China

**Keywords:** Automatic lesion segmentation, DCE MRI, Phase preservation denoising

## Abstract

We offer a framework for automatically and accurately segmenting breast lesions from Dynamic Contrast Enhanced (DCE) MRI in this paper. The framework is built using max flow and min cut problems in the continuous domain over phase preserved denoised images. Three stages are required to complete the proposed approach. First, post-contrast and pre-contrast images are subtracted, followed by image registrations that benefit to enhancing lesion areas. Second, a phase preserved denoising and pixel-wise adaptive Wiener filtering technique is used, followed by max flow and min cut problems in a continuous domain. A denoising mechanism clears the noise in the images by preserving useful and detailed features such as edges. Then, lesion detection is performed using continuous max flow. Finally, a morphological operation is used as a post-processing step to further delineate the obtained results. A series of qualitative and quantitative trials employing nine performance metrics on 21 cases with two different MR image resolutions were used to verify the effectiveness of the proposed method. Performance results demonstrate the quality of segmentation obtained from the proposed method.

## Introduction

Among a broad range of medical conditions [1–3], cancer is the most common and dangerous illness on the planet. Cancer is one of the leading causes of mortality among women across the world. Cancer develops when aberrant body cells begin to split and come into touch with healthy cells, causing them to become malignant. According to the World Health Organization’s (WHO) cancer research organizations (International Agency for Cancer Research (IARC) and American Cancer Society), 17.1 million new cancer cases were reported globally in 2018 [4]. Furthermore, breast cancer is said to be the world’s most frequent and fastest-growing cancer, affecting primarily women and caused by aberrant cell development around the breast lobules or ducts [5]. It is the second most frequent cancer in women that leads to mortality, after lung cancer [6]. The importance of early identification and therapy in improving the survival rate cannot be overstated. Mammography [7], ultrasound [8], biopsy CT scan [9] and MRI scan [10] are some medical imaging modalities used to diagnose breast cancer. Dynamic contrast-enhanced magnetic resonance imaging (DCE-MRI) is a type of imaging that produces three-dimensional high-resolution images with correct anatomical information that is not available with the other two commonly utilized imaging techniques, mammography and ultrasound. As a result, it is the most common and important method for diagnosing breast cancer. Due to the vast amount of data required, manual segmentation of various imaging techniques for suspected breast lesions is a tedious and time-consuming task [11, 12].

Many of the available segmentation algorithms are classified as supervised or unsupervised learning approaches [13–15]. The purpose of supervised learning is to create a trained model that can differentiate between distinct object labels [16–18]. Some popular supervised approaches in the literature are K-Nearest Neighbors (KNN) [19, 20], random forests (RF) [21], SVM [22], Bayesian, and deep learning, which is an advanced supervised technique [23–25], are some of the popular supervised approaches in the literature. Because supervised methods necessitate huge labelled datasets, they are a time-consuming and computationally expensive method of attaining an efficient outcome [26, 27]. Furthermore, supervised learning algorithms have limitations in terms of dataset quantity and quality, as well as the risk of overfitting.Due to limited patient numbers and time constraints, obtaining sufficient labelled data for practical clinical applications is difficult [28–30]. It is also likely that neighboring pixels take the same label or have a low number of connected components.

Unsupervised methods, on the other hand, require prior knowledge of required segmentation labels and rely on features like region, boundary texture, and image edges [31, 32]. Patterns (feature vectors) related to the same object are used in unsupervised approaches. These characteristics can be investigated and defined in accordance with needs. Moreover, unsupervised machine learning models learn from unlabeled data without human interaction. Once the desired outcomes have been obtained, they can be used as labelled data to train supervised learning models that create more durable outcomes. Unsupervised segmentation approaches are thought to be useful in dealing with more complex scenarios [33, 34]. Some important unsupervised segmentation techniques include: (1) clustering-based segmentation, such as fuzzy Cmeans and Kmeans [35, 36]; (2) edge-based segmentation, which relies on the fact that pixels in the background and foreground are distinct [37]; (3) region-based segmentation, such as region growing and region splitting-merging [38]. In these techniques, a seed selection is considered an important step. Energy-based segmentation is another essential and common segmentation technique, in which a result is generated by minimizing a constructed energy function [39]. Basbes [40] presents a chest wall delineation technique based on content analysis and Dijkstra for axial breast DCE-MRI. The authors want to simplify things by processing an on-risk zone that is better suited to optimisation and then segmenting lesions using a clustering technique. Live wire [41], active contour [42], level sets [43] and graph-based [44] are some of the various approaches that use the energy function. The energy function is constructed using boundary information in active contour and level set approaches, and the performance is dependent on the original curve. Graph is another major unsupervised segmentation technique [45, 46]. To develop a globally optimal solution, graph-based segmentation uses region and boundary information [47, 48]. Also, discrete optimization graph-based methods have become popular because of their performance in medical image segmentation [25, 49, 50]. Images are partitioned into numerous sub-graphs in this method, each of which represents a relevant object in the image. The image is first turned into a graph, with pixels, regions, or voxels representing the graph’s organized grid. Grid bias, which penalizes spatial directions and has a negative influence on computing, is one of the major downsides of such a system.

Traditional max flow algorithms use a graph-based formulation of max flow to provide a method for discrete optimization of an energy function. A discrete graph-based max flow problem can be directly mapped to the continuous optimization formulation of the continuous max flow model, yielding more accurate results [51, 52]. Continuous max-flow algorithms, on the other hand, have no stopping criteria and require a lot more repetition to reach convergence [53]. Unsupervised segmentation algorithms, on the other hand, encounter a number of challenges, particularly in MRIs [54, 55]. The existence of unavoidable noise during the acquisition of breast DCE-MRI has a significant impact on the accuracy of segmentation. Geometric aberrations and non-uniform light in tissues make segmentation even more difficult. Furthermore, during acquisition, patient movement may blur or even wipe away the border between the lesion and the background tissue. Without a multigrid and parallel implementation, employing a continuous max flow min cut algorithm on MRI images will provide a less efficient result due to existing noise and slow down convergence [56].

We present a fully automatic and unsupervised approach for accurate lesion segmentation to address the aforementioned problems. The framework incorporates a graph method (solved by formulating max flow and min cut problems in the continuous domain) with denoising methods and morphological operations. Although the continuous max flow (CMF) technique can reduce iterations while reducing computational load, the segmentation quality is shown to be highly dependent on the denoising step prior to execution. As a result, a good denoising technique is required prior to the segmentation process in order to remove noise while maintaining relevant information and structure. The first stage in lesion segmentation is to remove common background signals and improve the contrast of breast lesions. This phase is completed by registering the images and subtracting the pre- and post-contrast images. Following that, we employ phase preserved denoising and adaptive Weiner filtering to reduce noise and undesired artefacts while keeping crucial segmentation features like edge and boundary. To acquire the segmentation, this step is followed by a CMF algorithm. Finally, we delete the undesirable region from the generated image using a morphological technique to get the final result.

The rest of the paper is organized as follows. In Sect. 2, we go over the various ways for segmenting breast lesions in depth. The proposed lesion segmentation method is explained in Sect. 3. Section 4 analyses the experimental results and provides a detailed discussion, with the final section providing a concluding remark.

## Materials and methods

### Image subtraction after registration

The subtraction between pre- and post-contrast images is a critical and primary stage in our algorithm [57]. By removing common background signals, this method makes it easier to characterize lesions. With enhanced contrast in the breast lesion, the resulting image is acquired. The performance of subtraction, on the other hand, is dependent on the image pre and post images acquisition. A patient should not relocate from one imaging session to the next, which is not always possible. These unintentional movements cause a misalignment of image sequence [58]. As a result, image registration is required prior to image removal. Image registration is the geometrical transformation of one image to another. The normalized image is obtained from the subtraction of the pre contrast image from the post-contrast image after the registration.

1 $$\begin{aligned} I_{\text {sub}} = I_{\text {post}} - I_{\text {pre}} \end{aligned}$$

where $I_{\text {post}}$ and $I_{\text {pre}}$ are the post and pre contrast image sequence. $I_{\text {sub}}$ labels the image obtained from the subtraction of $I_{\text {pre}}$ from the $I_{\text {post}}$ after registration.

Let $I_{\text {reg}}$ is the registered image. With respect to the pre-contrast image, the post-contrast image is registered. The registration algorithm “imregtform” function in Matlab is used to correct the misalignment between the pre and post contrast images. The similarity metric and optimisation approach are also defined by “imregconfig”. The affine transformation and bicubic interpolation are used in the registration process.

2 $$\begin{aligned}&I_{\text {reg}} = registration(I_{\text {post}}) \end{aligned}$$

3 $$\begin{aligned}&I_{\text {sub}} = I_{\text {reg}} - I_{\text {pre}} \end{aligned}$$

#### Affine transformation model

Let us consider pre- and post-contrast DCE-MRI image as $I_{pre}$ and $I_{post}$ that are generated from the same imaging technique. $I_{reg}$ is the registered image. In our case, pre contrast image $I_{pre}$ is considered as the fixed image and the post-contrast image $I_{post}$ is the moving image. Also, *p* and *q* are coordinates for fixed and moving image. The relationship between $I_{reg}(p)$ and $I_{post}(p)$ is given as shown in Eq. 4

4 $$\begin{aligned} I_{reg}(p) = I_{post}(A(q)) \iff I_{post}(q) = I_{reg}(A^{-1}(p)) \end{aligned}$$

where *A* is the affine transformation. As demonstrated in the first, second, third, and fourth matrices, the affine transformation is the product of four geometric transformations: translation, rotation, scaling, and skew.

$$\begin{aligned} \begin{bmatrix} 1 &{} 0 &{} t_x \\ 0 &{} 1 &{} t_y \\ 0 &{} 0 &{} 1 \end{bmatrix} . \begin{bmatrix} \theta _c &{} -\theta _s &{} 0 \\ \theta _s &{} \theta _c &{} 0 \\ 0 &{} 0 &{} 1 \end{bmatrix} . \begin{bmatrix} 1 &{} k &{} 0 \\ 0 &{} 1 &{} 0 \\ 0 &{} 0 &{} 1 \end{bmatrix} . \begin{bmatrix} s_x &{} 0 &{} 0 \\ 0 &{} s_y &{} 0 \\ 0 &{} 0 &{} 1 \end{bmatrix} \end{aligned}$$

The dot product of these matrices is obtained as shown below:

$$\begin{aligned} \begin{bmatrix} s_x\theta _c &{} s_y(k\theta _c-\theta _s) &{} t_x \\ s_x\theta _s &{} s_y(k\theta _s+\theta _c) &{} t_y \\ 0 &{} 0 &{} 1 \end{bmatrix} \end{aligned}$$

where $t_x$ and $t_y$ are the shift of positive value towards left and up. $\theta$ is defined as the rotation which is measured in clockwise direction. *k* is a shear factor and $s_x, s_y$ are the change of scale in x and y direction respectively.

### Local phase-preserved denoising of DCE-MRI

Denoising DCE-MR images is a crucial step in the lesion segmentation process [59]. Denoising is a technique for transforming an image into a domain where the noise component may be easily identified. After that, the noise is removed and the image is turned into a noise-free image. Wavelet transformation is one of several denoising techniques that is thought to be particularly effective at distinguishing between signal and noise in an image. In addition, the image contains two critical pieces of information: magnitude and phase. It can be seen that the prior denoising process on breast DCE-MR images did not take this key information into account, namely phase information [60–62]. Not only for perception, but also for image improvement, phase information is critical.

A log Gabor wavelet filter is used in the phase preserved denoising approach. In DCE-MRI, the image is first deconstructed into amplitude and phase information at each point of slice. The observation reveals that the majority of the amplitude information is concentrated in the middle of the image, while the phase information is scattered throughout. It’s clear that amplitude or phase information alone isn’t enough to rebuild a noise-free image while keeping crucial visual properties. As a result, we develop a phase-preserved approach for DCE-MRI images of the breast that shrinks the amplitude information in various scaling factors and orientations.

Consider an image as a signal vector, *I*(*x*, *y*). (5) is the response vector for even symmetric (*Mne*) and odd symmetric (*Mno*) wavelets at scale *n*. At a wavelet scale *n*, the amplitude *An*(*x*) and phase *phin* are determined as (6) and (7), respectively.

5 $$\begin{aligned}{}[Re_n(x,y),Im_n(x,y)] = [I(x,y)\times M_n^e , I(x,y)\times M_n^o] \end{aligned}$$

where $Re_n(x,y)$,$Im_n(x,y)$ is real and imaginary part of complex valued frequency component.

6 $$\begin{aligned}A_n(x,y) = \sqrt{Re_n(x,y)^{2}+Im_n(x,y)^{2}} \end{aligned}$$

7 $$\begin{aligned}&\phi _n(x,y) = {\text{ atan2 }}(Im_n(x,y)/Re_n(x,y)) \end{aligned}$$

During denoising, a noise threshold is calculated at each wavelet scale, and the size of the filtered vector is decreased while the phase remains unchanged. As a result, the complex-valued wavelet response is used, in which the phase is kept while the amplitude is reduced across various wavelet sizes and orientations. Estimation of a signal can be reconstructed by summing the remaining even-symmetric filter response over all scales and orientations. The mean and variance of the Rayleigh distribution are used to estimate the noise threshold. The mean and variance of the Rayleigh distribution ${\mathbb {R}}$ is given by $\mu _{\mathbb {R}}$ and $\sigma _{\mathbb {R}}^2$ in (9).

8 $$\begin{aligned}&{\mathbb {R}}(x,y)= (x,y)/\sigma ^2 e^{-(x,y)^2/2\sigma ^2} \end{aligned}$$

9 $$\begin{aligned}\mu _{\mathbb{R}} = \sigma \sqrt{\pi /2}, \sigma _{\mathbb{R}}^{2} = \frac{4-\pi }{2} \sigma^{2} \end{aligned}$$

where $\sigma ^2$ is the scale parameter of the Rayleigh distribution. The noise threshold is calculated as,

10 $$\begin{aligned} \tau _1 = \mu _{\mathbb {R}}+ {\text{ c }} \sigma _{\mathbb {R}} \end{aligned}$$

where ${\text{ c }}$ is the standard deviation of the noise to be rejected. It has something to do with the perfect wave shape. The smaller the value of c, the more optimal the wave shape will be. The value of c was set to be fixed and equal to one.

To make a robust estimation, mean ($\mu_{\mathbb{R}}$) is replaced with the median ($\mathbb {M}$) response of Rayleigh distribution,

11 $$\begin{aligned} \mathbb {M} = \sigma \sqrt{-2 {\text{ ln }}(1/2)} \end{aligned}$$

where $\mathbb {M}$ labels median response. In each scale and orientation, the noise threshold is calculated and processed. Finally, the reconstructed image is obtained as $I_{\text{2 }}$.

### Adaptive wiener filtering

Because edges are key features during lesion segmentation, the edge-preserving denoising technique should be used. To smooth the image while keeping the edges, we use an adaptive Wiener filtering approach [63].

The adaptive Wiener filter is given by Eq. (12) [64]:

12 $$\begin{aligned} I_{\text {denoised}}(i,j) = m_{\text {f}} + \frac{\sigma _{\text {f}}^2 - v^2}{\sigma _{\text {f}}^2}(I_{\text {noisy}}(i,j)- m_{\text {f}}) \end{aligned}$$

where $m_{\text {f}}$ and $\sigma _{\text {f}}^2$ is the local mean and variance. $v^2$ is the average value of $\sigma _{\text {f}}^2$ across noisy image i.e. $I_{\text {noisy}}$. The computation of local mean and $m_{\text {f}}$ and variance $\sigma _{\text {f}}^2$ is provided in Eq. (13):

13 $$\begin{aligned} \begin{aligned} m_{\text {f}} = (XY)^{-1} \sum _{i,j \in M} I_{\text {noisy}}(i,j)\\ \sigma _{\text {f}}^2 = (XY)^{-1} \sum _{i,j \in M} (I_{\text {noisy}}^2(i,j)- m_{\text {f}}^2) \end{aligned} \end{aligned}$$

where *X* and *Y* are the horizontal and vertical arrays of pixels in the window mask.

### CMF based lesion segmentation

The Continuous Max Flow (CMF) [65] method is a graph-based methodology that has been demonstrated to be extremely effective in labelling key parts in an image. Consider the task of partitioning the $\Omega$ continuous image domain into two regions or labels: foreground and background. Source and sink are the two terminals.

**Fig. 1:**
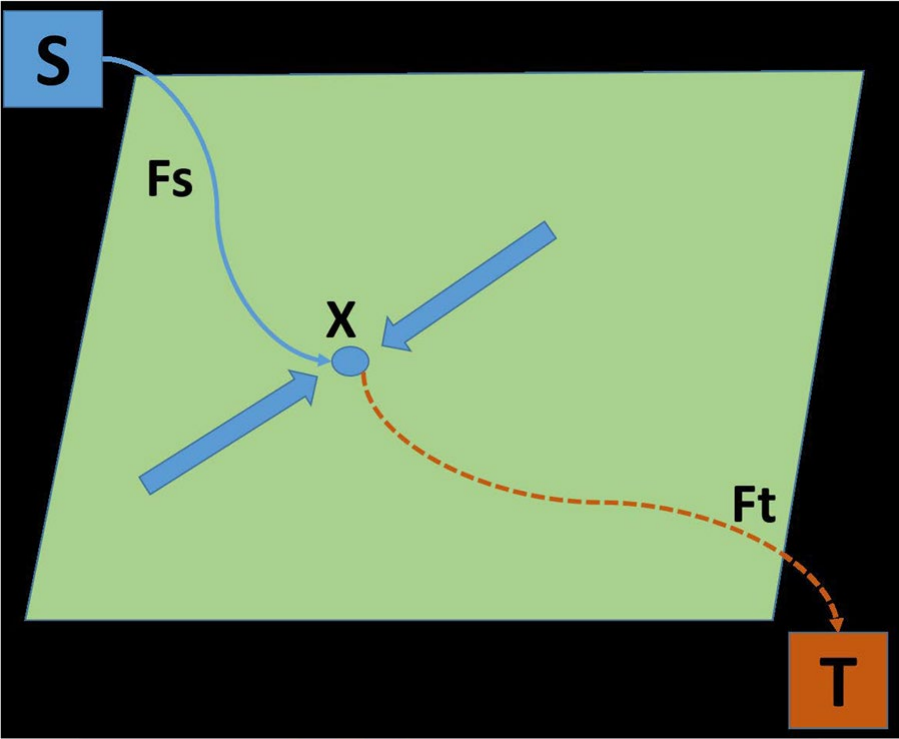
Continuous Max flow with two labels

There are three concerning flows: $F_s$, $F_t$ and *F* are the source, sink and spatial flow as shown in the Fig. 1. Let *x* be the image position and each image position $x \in \Omega$.

14 $$\begin{aligned} F_s(x) \le C_s(x), F_t(x) \le C_t(x), |F(x)| \le C(x) ; \forall x \in \Omega \end{aligned}$$

and the flows are conserved as

15 $$\begin{aligned} F_t - F_s + div F = 0; \forall x \in \Omega \end{aligned}$$

**Fig. 2:**
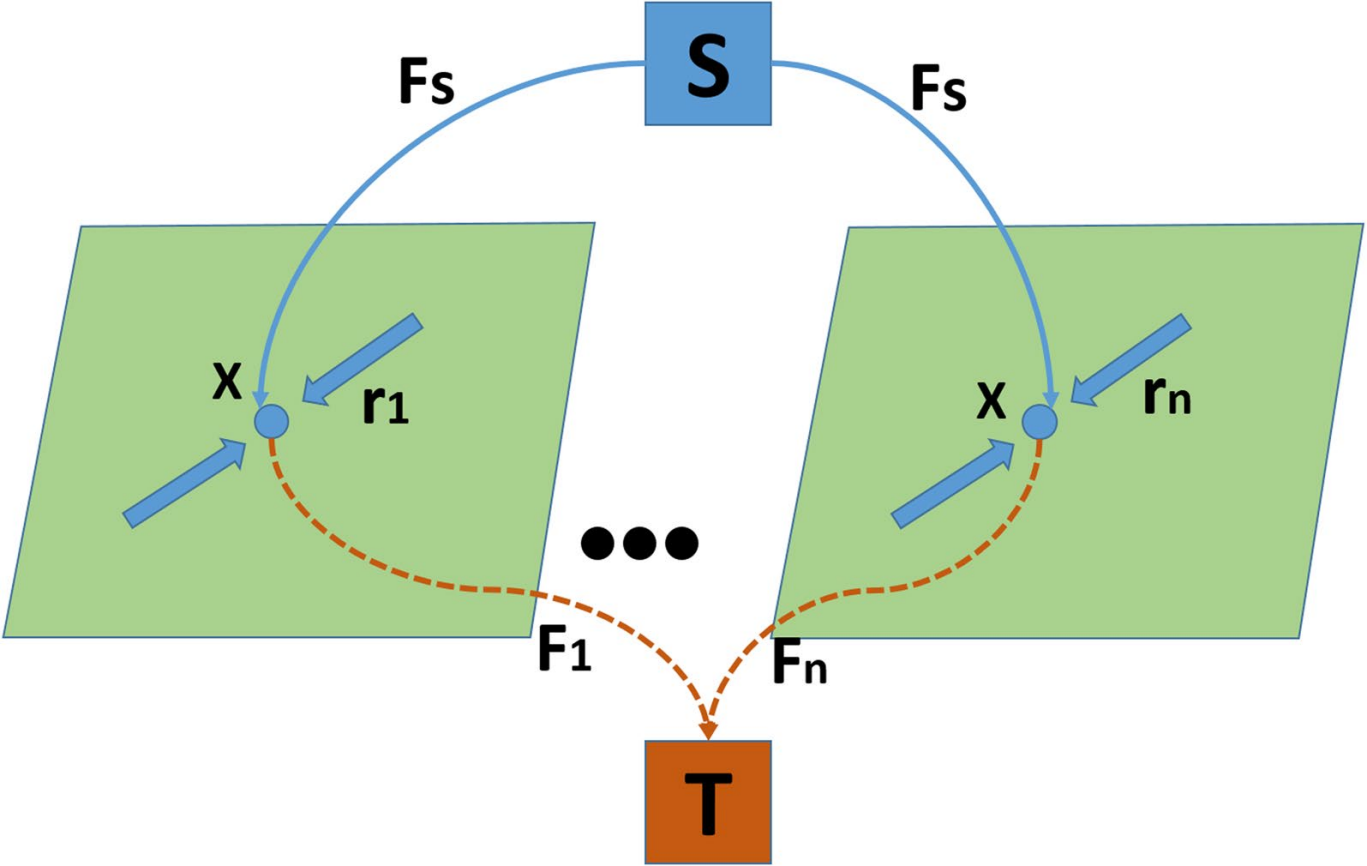
Continuous Max flow with *n* labels

As a result, for the total flow from source to sink for two labels, the max flow problem is given by16Consider the task of dividing the continuous image domain $\Omega$ into regions or labels with $i = 1 \ldots n$. The source, sink, and spatial flow are represented by $F_s(x), F_i(x) and r_i(x)$, respectively, as illustrated in Fig. 2. Let *x* be the image position, and $x \in \Omega$ be the image position. The *n* label max flow model of $\Omega _i$ is given in parallel, with $i = 1\ldots n$.

At each position $x \in \Omega$, $F_s(x)$ stream from *s* to *x* for each label $i=1\ldots n$ Hence, the source field is identical and there is no constraint for the source flow $F_s(x)$ for *n* label partition.

$F_i(x)$ and $r_i(x)$ are constrained by the capacities $\rho (L_i,x)$ and $C_i(x)$, $i=1\ldots n$.

The flow are conserved as

17 $$\begin{aligned} (div r_i - F_s + F_i)(x) = 0, i=1\ldots n \end{aligned}$$

Hence, the max flow problem for the total flow from source to sink for *n* labels is given by18The Potts model is regarded as a powerful image segmentation method. The mathematical expression for multi-region segmentation using the Potts model is provided as in the equation.19where $|\partial \Omega _i|$ is the perimeter of each disjoint sub domain $\Omega _i$, $i =1\ldots n$. $C_i(x)$, $i =1\ldots n$ is the cost of assigning the specified position $x \in \Omega$ to the region $\Omega _i$. The segmentation problem can be solved using convex relaxation potts model derived from Eq. 19 as shown in Eq. 2020where $u_i(x)$, $i=1\ldots n$ defines the function of the segmented region $\Omega _i$. *S* is the convex constrained set of $u(x)=(u_1(x),\ldots u_n(x))$

### Morphological operation

Morphological operators use a set function known as the structuring element to extract required structures from a image (SE) [66]. SE is selected according to the set of pixels of interest on the image. Erosion and Dilation are the two fundamental morphological operators used in this research. To eliminate all related components except the largest one, a combination of erosion and dilation is performed, and the lesion is then restored within the greatest achieved region.

Assume that $I_{MRimg}$ is a set of pixels from the original image, $I_{SE}$ is the structuring element, and $(\hat{I_{SE}})S$ is the reflection of $I_{SE}$ about its origin, followed by the shift of S [67]. Equation 21 shows how to get the dilation and erosion procedure.

21 $$\begin{aligned} & Dilation, I_{\text {MRimg}} \oplus I_{\text {SE}} = \{S|(\hat{I_{\text {SE}}})S \cap I_{\text {MRimg}}\}\\ & Erosion, I_{\text {MRimg}} \ominus I_{\text {SE}} = \{S|(\hat{I_{\text {SE}}})S \subseteq I_{\text {MRimg}}\} \end{aligned} $$

## Proposed lesion segmentation method

**Fig. 3:**
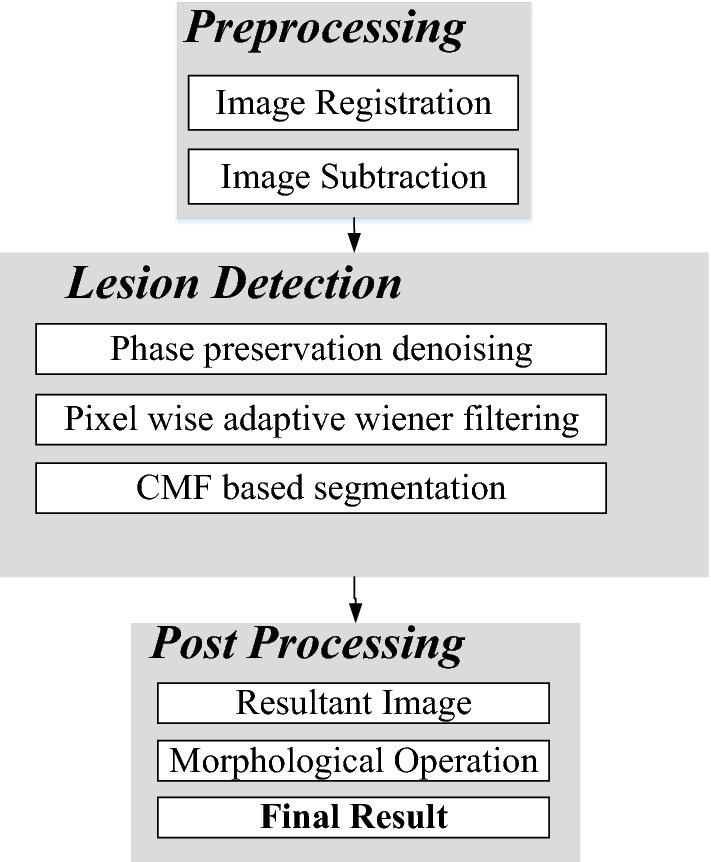
The proposed functional diagram of retinal vessel segmentation

The proposed segmentation approach consists of three steps: (1) image pre-processing, (2) lesion detection and (3) image post-processing as shown in Fig. 3.

### Image pre-processing

**Fig. 4:**
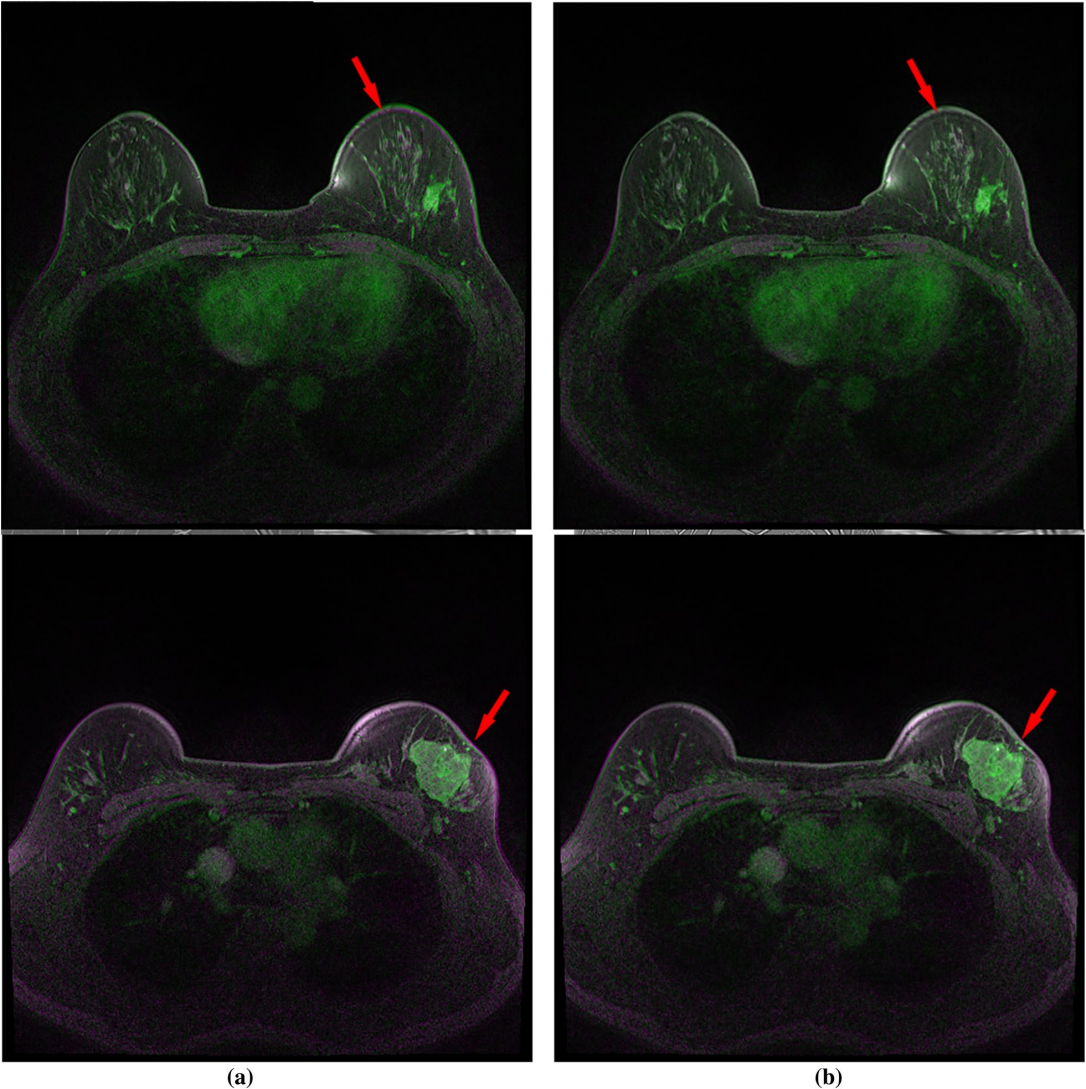
Illustration of image registration algorithm in DCE-MRI. (a) Pre-contrast image (b) Post contrast image

This procedure is utilized to provide a more enhanced normalized image, which makes it easier to notice the lesion. It’s done by subtracting the pre-contrast image from the post-contrast image obtained after the contrast agent is injected. Image registration should be done before the image subtraction. Image registration corrects the pre- and post-contrast image misalignment caused by inadvertent movement during imaging. Figure 4 shows the pre-contrast and post-contrast images.

**Fig. 5:**
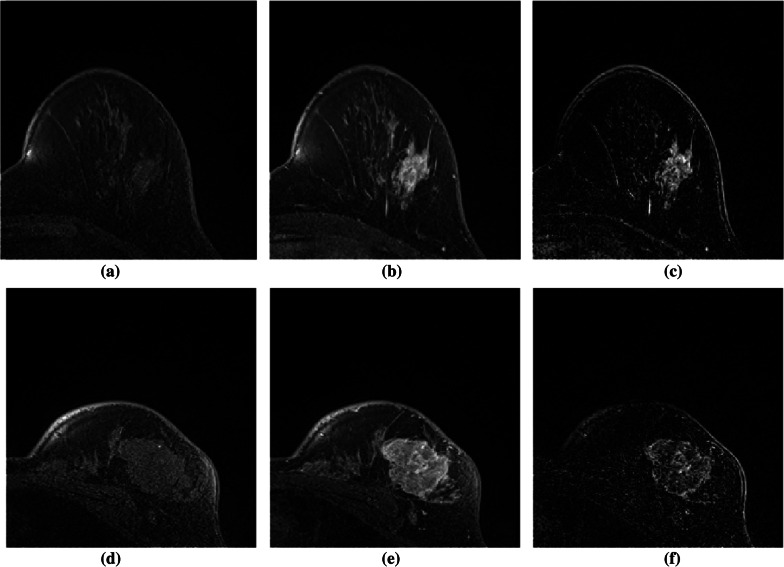
Subtraction of the pre-contrast from the post-contrast image. **a**, **d** pre-contrast image. **b**, **e** Post-contrast image. **c**, **f** The resultant image after subtraction of the pre-contrast image from Post-contrast image

The subtraction operation removes native T1 signal and hence the remaining enhancement is effective to accurately detect the lesion. This process is seen competent to the image where enhancement is critical to detect the complicated cysts. The figure illustrates the effectiveness of image subtraction. Figure 5a, d are the pre-contrast, (b), (e) are the post-contrast and (c), (f) are the resultant image after the image subtraction respectively.

### Lesion segmentation

DCE-MRI contains noise due to the fluctuations in the receiver coil and from the electrically conducting tissue. The presence of noise in the DCE-MRI image increases the complexity and leads towards the misinterpretation. It is necessary to remove this noise, minimize the new artifacts and preserve kinetic enhancement information and fine structural details. Therefore, following the pre-processing step, the phase preserved denoising method is applied. During lesion segmentation, it is also necessary to smooth the image while sharpening the edges. As a result, we use an adaptive Wiener filtering strategy following phase preserved denoising.

**Fig. 6:**
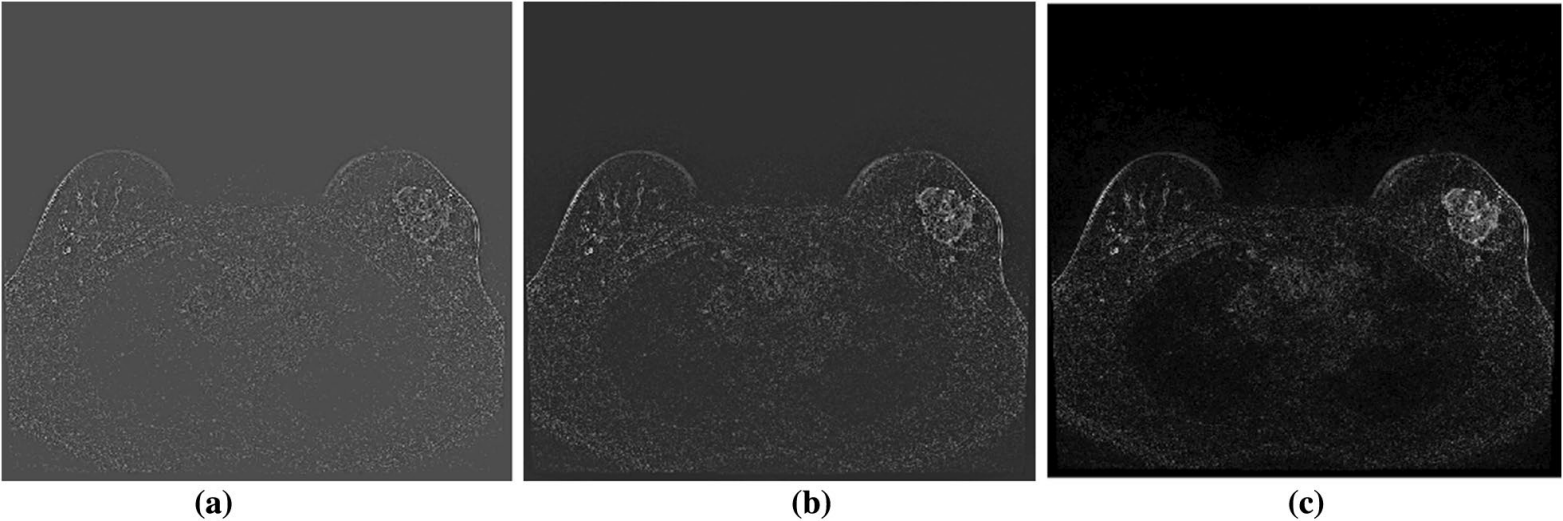
Filter responses of the DCE-MR images obtained according to different scaling factors. **a** Scaling factor of 1. **b** Scaling factor of 3. **c** Scaling factor of 8

The orientation and wavelet scaling factor are two crucial factors to consider when using the phase maintained denoising approach. Filter responsiveness to noise is high when the scaling factor is low. The filter response to noise will eventually be reduced as the scaling factor is increased. The scaling factor should be carefully chosen because a low scaling factor may treat beneficial information as noise and eliminate it. In addition, a high scaling filter may be poor at removing noise. We chose the scaling factor of 8 after multiple experiments and optimizations, which preserves fine structural details while increasing the contrast between the lesion and the background. The filter response of a DCE-MRI image via phase preserved denoising with different scaling factors is shown in Fig. 6.

**Fig. 7:**
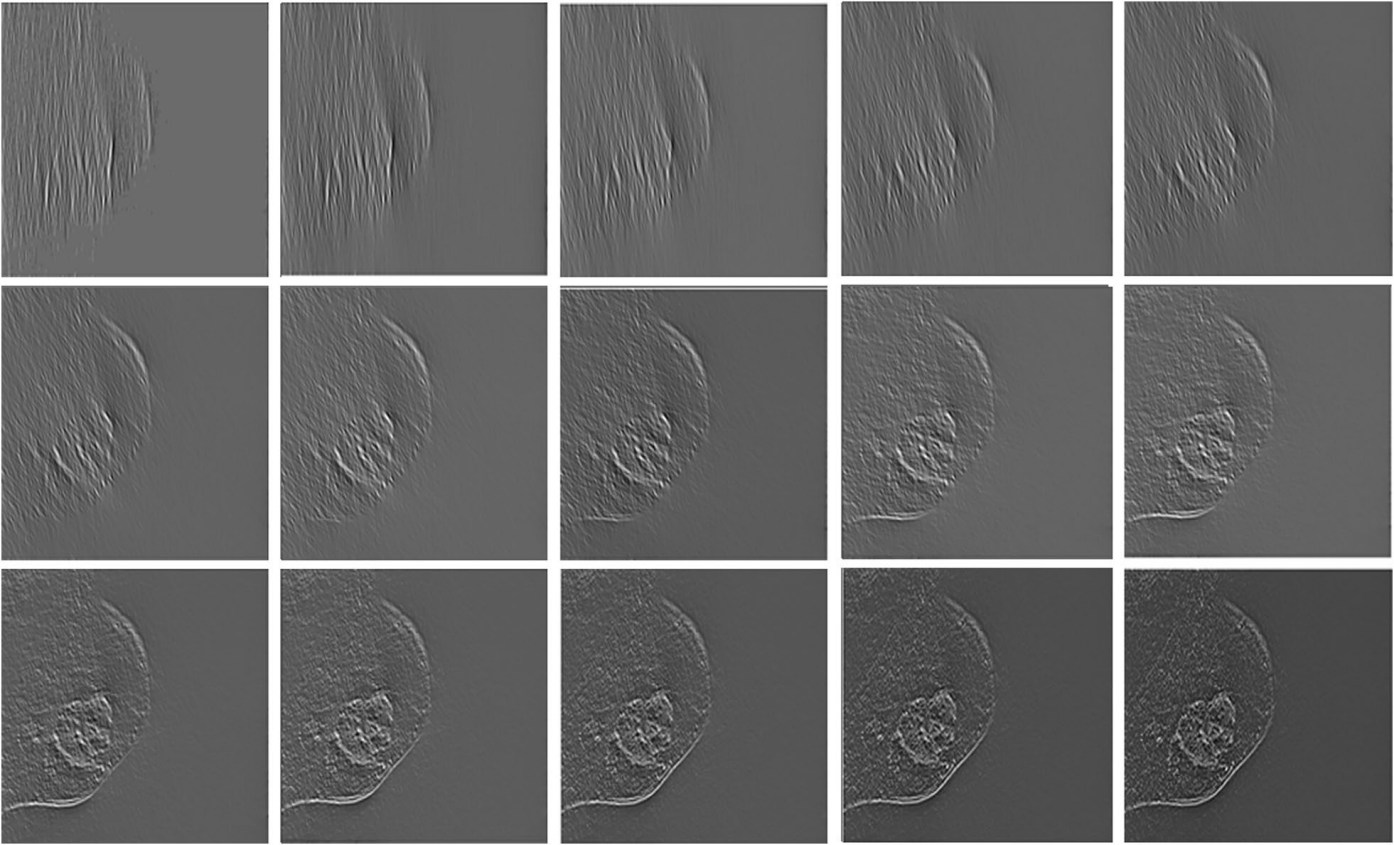
Illustration of phase preserved DCE-MRI after reconstruction with scaling factor of 2 and 15 orientations using Gabor wavelet filter

The generation of Gabor features with wavelet filters is the first step in the denoising process. The produced Gabor features are then involved with the DCE-MRI slices. As a result, a feature vector response is achieved. For example, if 2 scales and 15 orientations are considered, it generates 15 different feature vector responses of slices as shown in Fig. 7. As a result, summing responses across all sizes and orientations yields the final denoised image.

Although the image has been denoised to some amount, smoothing is essential before using CMF to obtain correct segmentation. Smoothing is required at this stage while maintaining edges and the border. Bilateral filtering is effective in removing noise from edges and boundaries, which are high-frequency zones. As a result, we used bilateral filtering to smooth the images while preserving edges and boundaries.

**Fig. 8:**
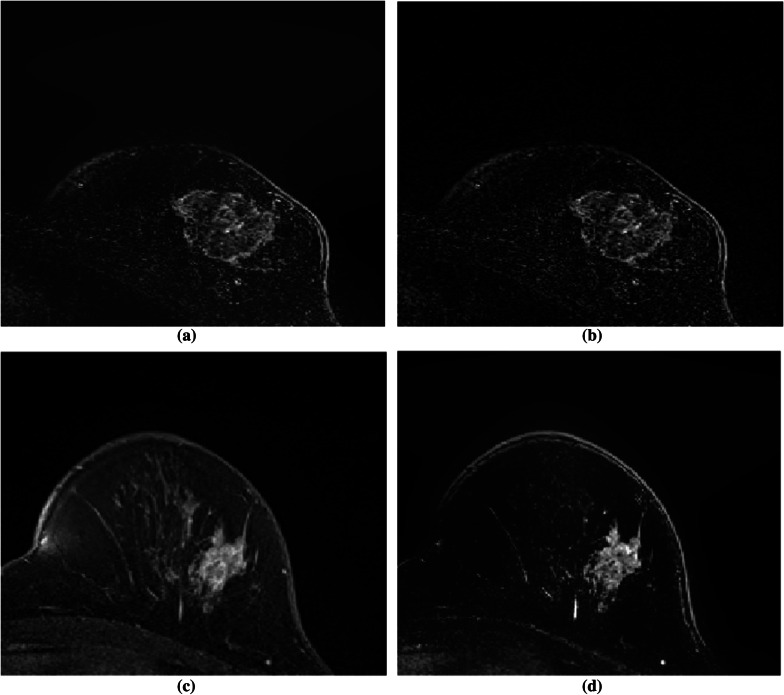
Images obtained with and without phase-preserved denoising and bilateral filtering are shown. Without utilising phase-preserved denoising and bilateral filtering, **a** and **c** are the final images after subtracting the pre-contrast from the post-contrast image. The resultant image after subtracting the pre-contrast from the post-contrast image with phase-preserved denoising and bilateral filtering is shown in (**b**) and (**d**)

Figure 8 show the image with or without using phase preserved denoising and bilateral filtering.

On the denoised MRI image acquired by phase preserving denoising, the continuous max-flow technique is applied. In the DCE-MRI, each pixel of each slice is coupled to the source *S* and sink *T* in the continuous plane at first. We also take into account that each pixel is linked to three different flows: source, sink, and spatial flow. The source flow is directed towards sink *T* from source *S*. The amount of interaction with its neighbouring pixels determines the spatial flow. For a noisy image with low SNR, the capacity values of all pixels would constrain solutions to a local minimum, preventing the global optimum from being determined. As a result, before applying the CMF approach, phase maintained denoise is employed to clear the image’s noise while preserving the image’s significant features. In addition, using bilateral filtering on a phase-preserved image will smooth it down while keeping the edges.

### Image post-processing

Based on the observed result from the earlier section, post-processing of the image is required. Morphological erosion and dilation operation are used to remove the boundary of edges. secondly, the nearby components are connected together and the biggest area among the connected component are searched and preserved. Rest of the areas are considered as noise and removed. The obtained segmented image are compared with manually drawn available ground truth image from the expert. Figures 10, 11, 12 show resultant images obtained after the post-processing. It is observed that post-processing plays a vital role to further precisely segment lesions.

### Data availability

The data acquired for this work was obtained from Zhongshan Hospital of Dalian University http://en.dlhospital.com/index-subject-detail-id-11.html. The datasets analyzed in this article are not publicly available, as the data contain potentially identifying or sensitive patient information. All the data has got ethical clearance. Upon request, the request for data should be sent to Dsqshenjing2002@163.com, Jing Shen, Affiliated Zhongshan hospital of Dalian University. Doctor Jing Shen assisted collecting the breast MRI data and drawing the tumour patterns manually. The detail on data acquisition technique is explained in the “Performance evaluation and results” section.

## Performance evaluation and results

### Data acquisition and evaluation criteria

The experiment is conducted on Windows 10 ($\times$64), with Intel Core i5 CPU, 2.9GHZ and 8GB RAM. We validate the proposed algorithm on the image generated from 1.5T scanner. The imaging parameters for DCE-MRI were: TR/TE = 4.5/1.8 ms, a matrix size = 512 $\times$ 512, with the number of signal averages set to 1, a field of view of 30 cm, and a slice thickness of 1.5 mm. The gray-level range of MRIs is 0–255. There are total 23 cases in which 19 cases with the size of 512 $\times$ 512 $\times$ 96 and 4 cases with the size of 480 $\times$ 480 $\times$ 160. All cases have one pre-contrast and 4 post-contrast imaging frames were acquired. Ground truth images are available for all the cases, which are manually labeled by qualified doctors. The result for the lesion segmentation is acquired from, 2464 scans of 23 cases. For the experiment, we have divided the images into two groups : G1 and G2. G1 includes images with resolution of 512 $\times$ 512 $\times$ 96 and G2 with resolution of 480 $\times$ 480 $\times$ 160.

The performance of a denoised image is first illustrated by calculating the peak signal-to-noise ratios before and after phase conserved denoising (PSNR). Furthermore, the quantitative assessment of the proposed algorithm is tested with nine metrics: accuracy (Acc), sensitivity (Se) or Recall, Specificity (Sp), precision (P), the error rate (ER), Volumetric Similarity (Vs), DICE coefficient (DSC), Jaccard index (Jc) and AUC from receiving operating characteristics (ROC) curve. These parameters are determined using a pixel-based classification technique, in which each pixel on a DCE-MRI slice is categorized as a lesion or a background. There are four possible combinations in the pixel-based classification technique: two classifications and two misclassifications. True positive (TP) and true negative (TN) pixels are those that have been accurately identified during categorization. The term misclassification refers to the false positive (FP) and false negative (FN) which are incorrectly identified as a lesion. SG signifies the segmentation’s obtained from the proposed methods and GT signify the ground truth which is manually segmented. These metrics are defined in the following equations.

22 $$\begin{aligned}&\text {Acc} = \frac{\text {TP}+\text {TN}}{\text {TP}+\text {FP}+\text {TN}+\text {FN}} \end{aligned}$$

23 $$\begin{aligned}&\text {Se} = \frac{\text {TP}}{\text {TP}+\text {FN}} \end{aligned}$$

24 $$\begin{aligned}&\text {Sp} = \frac{\text {TN}}{\text {TN}+\text {FP}} \end{aligned}$$

25 $$\begin{aligned}&\text {P} = \frac{\text {TP}}{\text {TP}+\text {FP}} \end{aligned}$$

26 $$\begin{aligned}&\text {ER} = \frac{\text {FP+FN}}{\text {TP}+\text {FP}+\text {TN}+\text {FN}} \end{aligned}$$

27 $$\begin{aligned}&\text {Vs} = 1 - \frac{\text {FN} - \text {FP}}{2 \times \text {TP} + \text {FP}+ \text {FN}} \end{aligned}$$

28 $$\begin{aligned}&\text {DSC} = \frac{2(\text {SG} \cap \text {GT})}{\text {SG} + \text {GT}} \times 100\% \end{aligned}$$

29 $$\begin{aligned}&\text {JC} = \frac{(\text {SG} \cap \text {GT})}{\text {SG} \cup \text {GT}} \times 100\% \end{aligned}$$

Acc is the ratio of the total number of correctly classified pixels to the total number of pixels in an image. The metrics Se and Sp are obtained from the proportion of positively and negatively recognised pixels in the ground truth image. The ratio of accurately predicted positive observations to total expected positive observations is denoted by the letter P. P denotes repeatable measurements, even if the value is outside of the acceptable range, distinguishing it from accuracy. The mis-classification rate (ER) is a metric error rate that assesses the frequency of incorrect predictions. When the ER is near to 0 and positive, it is considered outstanding. The volume of the segments that suggested similarity is measured by volumetric similarity (Vs). It is calculated by dividing the absolute difference by the sum of the comparative volumes. DSC is the overlap based metric that measures the similarity between segmented OD via automatic and manual method. To further verify the efficiency of the proposed algorithm, we calculated a metric known as JC. This metric is the similarity measure related to the Jaccard index which measures the overlap between automatically and manually segmented OD. We employ the AUC metric, which is derived from the receiving operating characteristics (ROC) curve, to assess the trade-off between Se and Sp. The curve is displayed with a false positive rate (1-Sp) on the x-axis and a true positive rate (Se) on the y-axis using varying threshold values within a certain interval to create this non-parametric performance measurement. A result of greater than 90% is considered excellent, and the ROC curve is deemed ideal when it is closer to the top left corner, which provides a perfect value, i.e. 1.

### Results and discussion

The DCE-MRI image is noisy in its original form. The algorithm’s performance is affected by the segmentation of the lesion from the noisy image. As a result, phase preserved denoising is employed to remove the image’s undesired noise and artefacts. The image enhancement is visible in Fig. 8 and can be tested by calculating the PSNR value before and after denoising, as shown in Table 1. We separated the entire number of images into two groups (G1 and G2) and calculated the PSNR value acquired before and after denoising because the data set contains images with two resolutions. As shown in Table 1, the PSNR value in both groups has improved significantly.

**Fig. 9:**
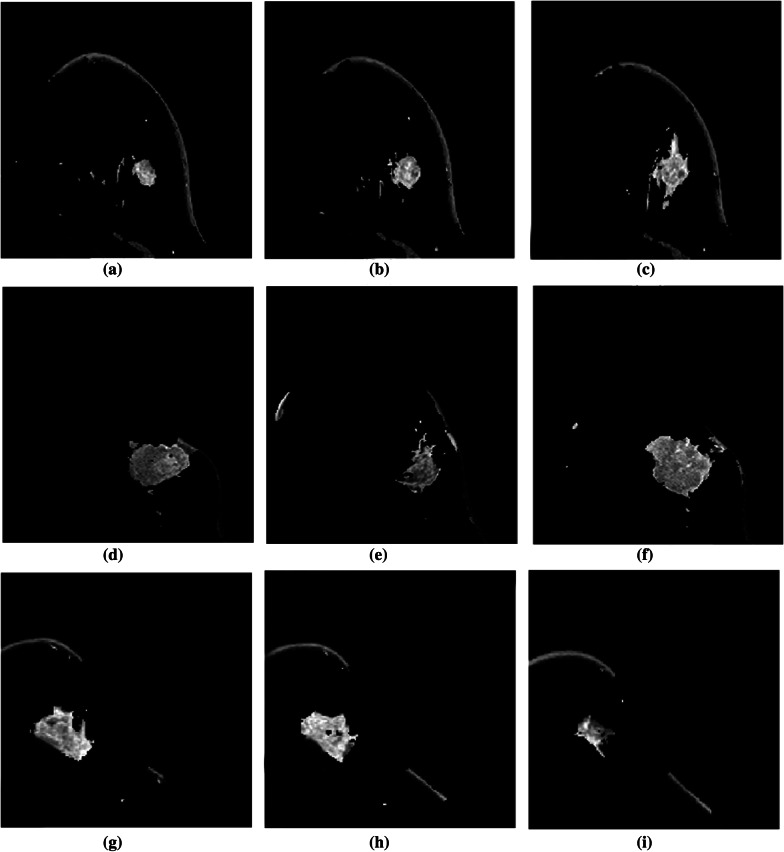
Illustration of resultant lesion segmentation obtained by using the proposed method before post-processing. Each row (a)-(b)-(c), (d)-(e)-(f), and (g)-(h)-(i) are the lesion segmented from slices from the same MR images

**Fig. 10:**
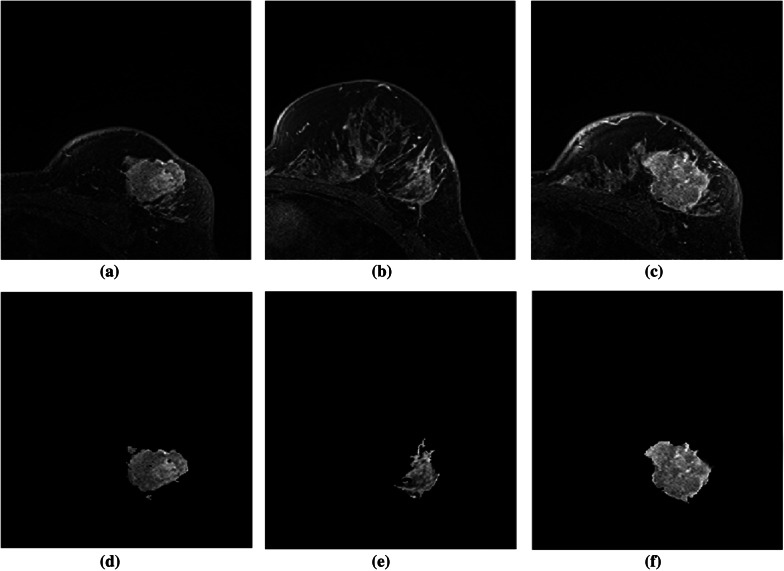
Illustration of resultant lesion segmentation obtained by using the proposed method after post-Processing. First row (**a**, **b**, **c**) represents the original image and second row (d, e, f)represents the final result i.e segmented lesions

**Fig. 11:**
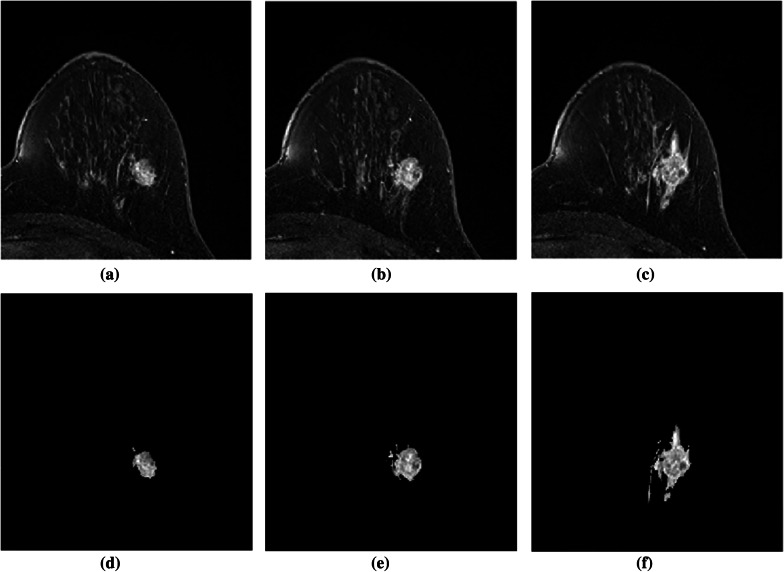
Illustration of resultant lesion segmentation obtained by using the proposed method after post-processing. First row (a, b, c) represents the original image and second row (**d**, **e**, **f**) represents the final result i.e. segmented lesions

**Fig. 12:**
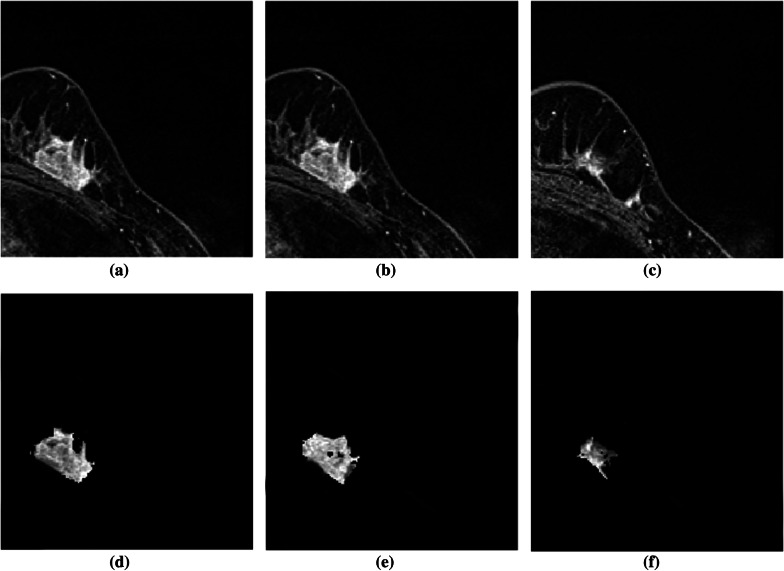
Illustration of resultant lesion segmentation obtained by using the proposed method after post-Processing. First row (**a**, **b**, **c**) represents the original image and second row (**d**, **e**, **f**)represents the final results i.e segmented lesions

**Fig. 13:**
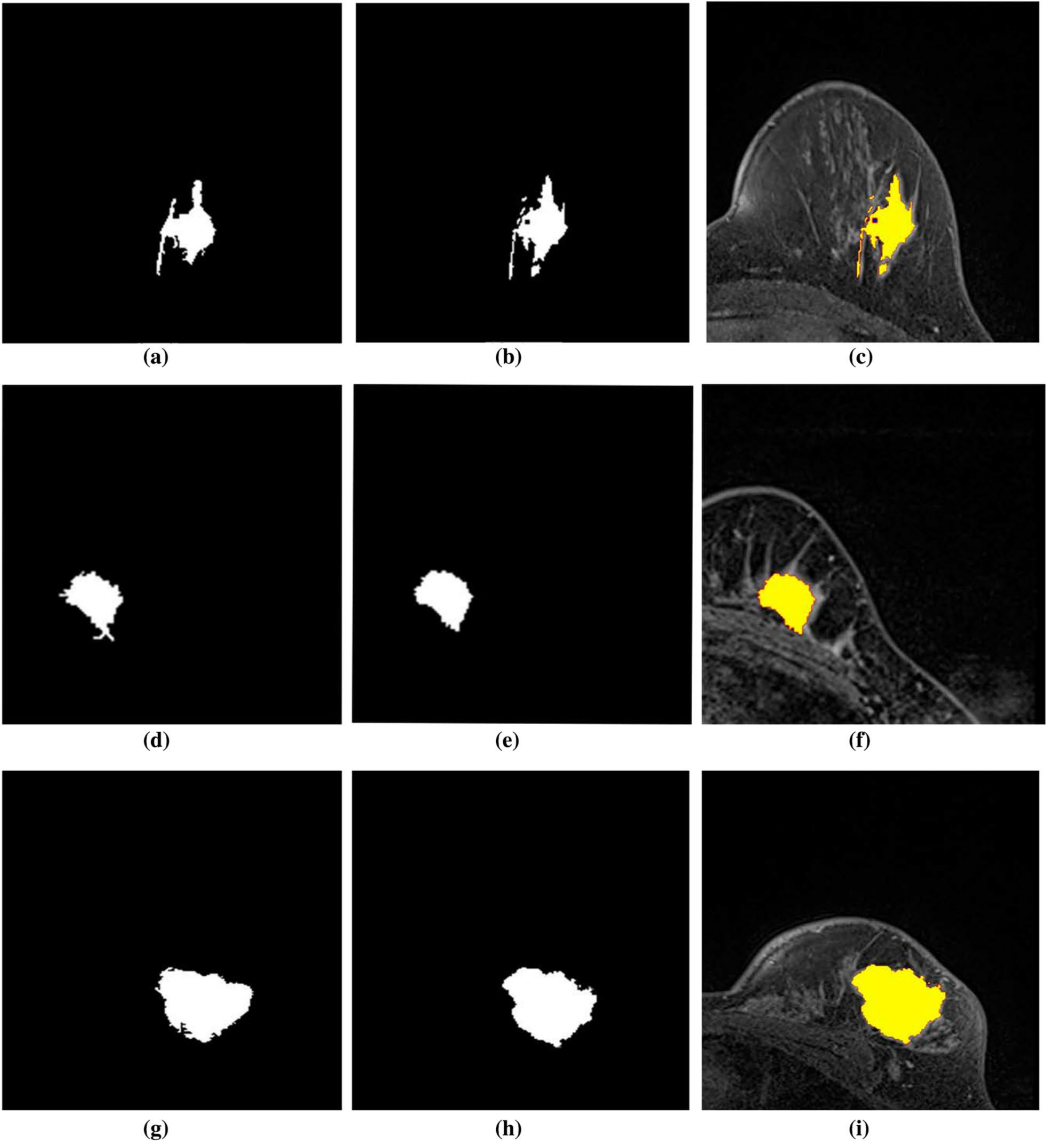
Results of lesion segmentation on the MRI images with different levels of BD and different breast shapes. The images in the first column are the manually segmented ground truth images. Similarly, second and third columns are the automatically segmented results with the proposed method and its mask on the original image to visually inspect the accuracy

The outcomes of the segmentation can be seen graphically. The proposed method’s subsequent lesion segmentation is shown in Fig. 9. Although the suggested method is capable of efficiently segmenting the lesion, some undesirable areas are present in the image, necessitating extra processing. Hence, the post-processing step is carried out in the obtained resultant image. Figures 10, 11 and 12 show how the post-processing procedure can remove the majority of the undesired portions from the final photos. When compared to the ground-truth image, the proposed method shows that it is capable of efficiently segmenting the lesion as shown in Fig. 13. A skilled radiologist manually segmented the ground truth image as shown in Fig. 13a, d and g. The ultimate outcome of the proposed method is shown in Fig. 13b, e, and h. The overlap between the lesion region in the original image and the result achieved using the suggested method is shown in Fig. 13c, f and i. The results reveal that the suggested method is capable of accurately segmenting the lesion region, which is supported by quantitative analysis as shown in Table 2. We used nine parameters to evaluate the effectiveness of the suggested work in this research.

**Table 1:** Comparison of PSNR values before and after the phase preserved denoising

Dataset	Average PSNR	
	Subtracted image	After denoising
G1	$21.36 \pm 0.7$	$32.54 \pm 0.42$
G2	$20.82 \pm 0.21$	$34.19 \pm 0.53$

**Table 2:** Quantitative comparison of performance of lesion segmentation using the proposed method with the ground-truth image

	Acc	Se	Sp	P	ER	Vs	DICE	JC	Auc
Cases (G1)									
1	0.9933	0.9081	0.9968	0.9242	0.0023	0.9912	0.9161	0.8451	0.98
2	0.9921	0.9152	0.9841	0.9365	0.0069	0.9878	0.909	0.8569	0.97
3	0.9789	0.9231	0.9799	0.9388	0.0052	0.9921	0.9256	0.8654	0.96
4	0.9888	0.9012	0.9969	0.9219	0.0042	0.9874	0.9158	0.8475	0.97
5	0.991	0.897	0.9912	0.9127	0.0035	0.9789	0.92	0.8489	0.97
6	0.9699	0.8999	0.9879	0.9099	0.0058	0.9856	0.909	0.8585	0.98
7	0.9956	0.9258	0.9799	0.9123	0.0069	0.9956	0.9158	0.8741	0.99
8	0.9874	0.9265	0.9936	0.9223	0.0063	0.9961	0.9146	0.8461	0.99
9	0.9715	0.9241	0.9752	0.9234	0.0042	0.9816	0.9256	0.849	0.96
10	0.9865	0.9125	0.9858	0.9145	0.0043	0.9777	0.9241	0.851	0.97
11	0.9784	0.8812	0.9912	0.9156	0.0078	0.9713	0.9174	0.8479	0.98
12	0.9953	0.8845	0.9873	0.9215	0.0061	0.9782	0.916	0.859	0.99
13	0.9741	0.9178	0.9932	0.93	0.0039	0.9745	0.9292	0.8513	0.96
14	0.9799	0.9167	0.9889	0.92	0.004	0.9878	0.902	0.8467	0.97
15	0.9898	0.909	0.9798	0.912	0.0043	0.9923	0.9088	0.8419	0.98
16	0.9632	0.8821	0.9712	0.909	0.004	0.9891	0.9087	0.8521	0.98
17	0.9787	0.8928	0.9912	0.9097	0.0047	0.9858	0.9087	0.8484	0.99
18	0.9963	0.9181	0.9963	0.9312	0.0068	0.9799	0.9145	0.8546	0.96
19	0.9879	0.9099	0.9874	0.9012	0.0054	0.9889	0.9191	0.861	0.97
**Avg**	0.9845	0.9076	0.9877	0.9195	0.0051	0.9856	0.9158	0.8525	0.975
Cases (G2)									
1	0.9674	0.9191	0.9926	0.909	0.0052	0.9826	0.9193	0.8321	0.97
2	0.9874	0.9221	0.9874	0.9258	0.0045	0.9948	0.9201	0.8596	0.99
3	0.9742	0.8989	0.9858	0.9123	0.0054	0.9797	0.9078	0.8679	0.98
4	0.9931	0.9182	0.9745	0.9097	0.0068	0.9889	0.9183	0.8545	0.97
**Avg**	0.9805	0.9145	0.9850	0.9142	0.0054	0.9865	0.9163	0.8535	0.9775

The quantitative result achieved from the suggested method when compared to the ground-truth image is shown in Table 2. In every situation, we’ve had outstanding outcomes. In both groups, the average result is very similar when compared with the ground-truth image. The most of the results that we achieved are above 90% which shows that the performance of the proposed algorithm is excellent.

**Table 3:** Quantitative comparison of performance of lesion segmentation using the proposed method with the recently developed other approaches

	Acc	DSC	JC	Sp	Se	Auc
Conte et al. (2020)	$\times$	$\times$	$\times$	$\times$	0.75	0.70
Vogl et al. ( 2019)	$\times$	0.665	$\times$	0.93	0.94	0.97
Li et al. (2018)	$\times$	0.89	0.81	$\times$	0.88	$\times$
Rasti et al. (2017)	0.9639	$\times$	$\times$	0.9487	0.9773	$\times$
Jayender et al. (2014)	0.9	0.77	$\times$	$\times$	1	$\times$
Darryl et al. (2014_	$\times$	0.76	$\times$	$\times$	$\times$	$\times$
Marrone et al. (2013)	0.98	$\times$	$\times$	0.989	0.71	$\times$
Proposed method (G1)	0.9845	0.9158	0.8525	0.9873	0.9076	0.975
Proposed method (G2)	0.9805	0.9163	0.8535	0.985	0.9145	0.977

x = Not available; G1 = Group 1; G2 = Group 2

The experiments show that the results obtained from the proposed methods when compared with the results obtained from the recent methods outperform or highly comparable, as shown in Table 3. It is observed that Acc, Sp, Vs, and AUC obtained from the proposed method are above 95%, proving the effectiveness of the proposed algorithm. Also, in terms of overlapping metrics (DSC and JC), the obtained result outperforms or highly comparable with the existing methods with an average of 91.63% and 85.35% respectively. When comparing with the result obtained from the recently proposed method, Accuracy was observed to be better than all the other methods except Marrone et al. 2013. However, the result is highly comparable. The result obtained from the proposed method outperforms all the existing methods in terms of DSC and JC with an average value of 91.63% and 85.35%.

The task of segmenting a lesion from a breast DCE-MR image is significant and difficult. To obtain the previously indicated level of accuracy, we conducted multiple experiments before obtaining at the presented solution. The lesion appears in varying shapes and intensities in distinct DCE-MRI slices. Furthermore, due to unattended movement of the object, DCE-MRI images are packed with sounds during image collecting. To solve this issue, we came to the conclusion that image registration is required as the first step. Furthermore, geometric distortion and non-uniform lighting in the tissues are reported to complicate the segmentation procedure. We used the phase preserved denoising technique in the registered image, followed by pixel-wise adaptive Wiener filtering to maintain the sharp edges, to preserve the majority of the image’s relevant information while removing the noise. The significant section of the image is then labelled using the graph-based technique, i.e. CMF. This strategy has been shown to be successful in solving the segmentation problem while allocating the minimum parameter. Also, reducing the iteration time will assist in obtaining the faster segmentation. It is observed that the efficiency of this framework depends upon the denoising process prior to the application of the CMF algorithm. The CMF algorithm is experimented with or without using the pre-processing step. The experiment shows that the outcomes of CMF algorithm with the pre-processing steps are accurate in segmenting the lesion region. Without the pre-processing stage, the outcome contains a lot of undesirable areas, especially near the lesion.

The studies reveal that combining the CMF algorithm with phase preserved denoising produced a final image that encompassed the majority of the lesion region. However, the image still includes some undesired area in the image, which is displayed in Fig. 9. As a result, we’ve introduced a post-processing step to eliminate the undesired component. Initially, morphological dilation with a disc-shaped structuring element with a 5 pixel radius was used. This technique provides for a 5 pixel growth in all directions from the edges. The not connected lesion will be preserved as a result of this procedure, especially in the vicinity of the lesion. The procedure is then repeated, with each connected component being searched and the largest region being saved. To produce the final lesion segmentation, the convolution procedure is performed with the dilated image and the resulting image.

## Conclusions

We suggested an automatic and quick lesion segmentation method from breast DCE-MRI data in this work. As a pre-processing step, image registration is used before image subtraction. In addition, the pre-processed image is subjected to phase preserved denoising and adaptive Wiener filtering, followed by the CMF algorithm (a graph-based approach). Finally, post-processing is used to remove any undesired noises that remain, excluding the lesion. This framework has been tested with 23 different DCE-MRI cases with resolutions. In terms of nine metrics, the quantitative analysis reveals a significant improvement in segmentation quality when compared to recent segmentation techniques. Furthermore, the suggested unsupervised method requires no prior information and may be used with most medical images with little parameter changes. We will test the proposed framework with a big dataset in the future, and the segmentations that are acquired can be utilized as a label for the classification of various types of cancers.
